# Differential risk factor profile and neuroimaging markers of small vessel disease between lacunar ischemic stroke and deep intracerebral hemorrhage

**DOI:** 10.1177/17562864241253901

**Published:** 2024-05-23

**Authors:** Yajun Cheng, Maria del C. Valdés Hernández, Mangmang Xu, Shuting Zhang, Xiaohua Pan, Baoqiang An, Joanna M. Wardlaw, Ming Liu, Bo Wu

**Affiliations:** Department of Neurology, West China Hospital, Sichuan University, Chengdu, China; Centre for Clinical Brain Sciences, UK Dementia Research Institute, University of Edinburgh, Edinburgh, UK; Department of Neurology, West China Hospital, Sichuan University, Chengdu, China; Department of Neurology, West China Hospital, Sichuan University, Chengdu, China; Department of Neurology, Baotou Eighth Hospital, Baotou, China; Department of Neurology, Baotou Central Hospital, Baotou, China; Center of Cerebrovascular Disease, Inner Mongolia AeroSpace Hospital, Hohhot, China; Centre for Clinical Brain Sciences, UK Dementia Research Institute, University of Edinburgh, Edinburgh, UK; Department of Neurology, West China Hospital, Sichuan University, No. 37 Guo Xue Xiang, Chengdu 610041, China; Department of Neurology, West China Hospital, Sichuan University, No. 37 Guo Xue Xiang, Chengdu 610041, China

**Keywords:** cerebral small vessel disease, deep perforator arteriopathy, intracerebral hemorrhage, lacunar ischemic stroke, risk factor

## Abstract

**Background:**

Lacunar ischemic stroke (LIS) and deep intracerebral hemorrhage (dICH) are two stroke phenotypes of deep perforator arteriopathy. It is unclear what factors predispose individuals with deep perforator arteriopathy to either ischemic or hemorrhagic events.

**Objectives:**

We aimed to investigate risk factors and neuroimaging features of small vessel disease (SVD) associated with LIS *versus* dICH in a cross-sectional study.

**Methods:**

We included patients with clinically presenting, magnetic resonance imaging-confirmed LIS or dICH from two tertiary hospitals between 2010 and 2021. We recorded vascular risk factors and SVD markers, including lacunes, white matter hyperintensities (WMH), perivascular spaces (PVS), and cerebral microbleeds (CMB). Logistic regression modeling was used to determine the association between vascular risk factors, SVD markers, and stroke phenotype. We further created WMH probability maps to compare WMH distribution between LIS and dICH.

**Results:**

A total of 834 patients with LIS (mean age 61.7 ± 12.1 years) and 405 with dICH (57.7 ± 13.2 years) were included. Hypertension was equally frequent between LIS and dICH (72.3% *versus* 74.8%, *p* = 0.349). Diabetes mellitus, hyperlipidemia, smoking, and prior ischemic stroke were more associated with LIS [odds ratio (OR) (95% confidence interval (CI)), 0.35 (0.25–0.48), 0.32 (0.22–0.44), 0.31 (0.22–0.44), and 0.38 (0.18–0.75)]. Alcohol intake and prior ICH were more associated with dICH [OR (95% CI), 2.34 (1.68–3.28), 2.53 (1.31–4.92)]. Lacunes were more prevalent in LIS [OR (95% CI) 0.23 (0.11–0.43)], while moderate-to-severe basal-ganglia PVS and CMB were more prevalent in dICH [OR (95% CI) 2.63 (1.35–5.27), 4.95 (2.71–9.42)]. WMH burden and spatial distribution did not differ between groups.

**Conclusion:**

The microangiopathy underlying LIS and dICH reflects distinct risk profiles and SVD features, hence possibly SVD subtype susceptibility. Prospective studies with careful phenotyping and genetics are needed to clarify the mechanisms underlying this difference.

## Introduction

Cerebral deep perforator arteriopathy, also termed arteriolosclerosis, is a pathological process that predominantly affects the small perforating arterioles supplying the deep brain structures.^1^ It is a prevalent form of cerebral small vessel disease (SVD), manifesting as lacunar ischemic stroke (LIS) and deep intracerebral hemorrhage (dICH).^2^ The two stroke phenotypes account for 25% of all ischemic strokes and most ICH, respectively.^3^ Although both LIS and dICH are considered as consequences of deep perforator arteriopathy, our knowledge of the mechanisms underlying the two distinct stroke phenotypes remains limited. A more detailed understanding of the factors predisposing individuals with deep perforator arteriopathy to either ischemia or hemorrhage is required to elucidate pathophysiology and to inform preventive strategies.

Several studies have compared vascular risk factor profiles as indicators of underlying vasculopathy between patients with LIS and dICH,^4–20^ but the results were inconclusive due to limitations such as small sample sizes, inconsistent definitions of risk factors, suboptimal stroke subtype classification, and inclusion limited to subjects with hypertension (summarized in Supplemental Table S1); warranting an update.

Beyond vascular risk factors, magnetic resonance imaging (MRI) markers of SVD, including lacunes, white matter hyperintensities (WMH), perivascular spaces (PVS), and cerebral microbleeds (CMB), show the tissue effects of the underlying perforating arteriole pathology.^21^ Investigating these SVD markers holds promise for understanding the clinical course of deep perforator arteriopathy. While each individual SVD marker has been associated with an increased risk of stroke,^22^ their relative contribution to the propensity for LIS *versus* dICH is understudied. Current research suggests that CMB are more prevalent in dICH than LIS,^13,19,20^ but the relationship between other SVD markers and stroke phenotype is not well established.

We aimed to comprehensively assess vascular risk factors and SVD markers in patients presenting clinically with symptomatic LIS or dICH confirmed by MRI, to identify if any factors predispose to either LIS *versus* ICH.

## Methods

This study was performed in accordance with the Strengthening the Reporting of Observational Studies in Epidemiology guidelines for reporting observational research.^23^

### Patients

We identified patients with LIS from a prospective stroke registry that enrolled patients with acute ischemic stroke admitted within 7 days of onset at two large tertiary hospitals in China: West China Hospital between 2010 and 2020 and Baotou Central Hospital between 2018 and 2021. Patients routinely underwent MRI, vascular imaging, echocardiography, and electrocardiography. LIS was defined as clinical symptoms relevant to lacunar stroke and a recent small subcortical infarct measuring ⩽20 mm in axial diameter in a relevant brain region for the symptoms on diffusion-weighted imaging (DWI). We excluded patients with significant (>50%) stenosis of the ipsilateral extracranial or intracranial artery, cardioembolic source of embolism (e.g. atrial fibrillation), or other specific causes of stroke (e.g. arterial dissection). Patients were excluded if they were <18 years, had cortical infarct, liver cirrhosis, malignant tumor, hematological or autoimmune diseases.

We screened ICH patients with available MRI admitted to West China Hospital between 2010 and 2020. ICH with secondary causes like vascular malformation, tumor, Moyamoya, and hemorrhagic transformation of ischemic stroke were excluded. Our clinical routine is to perform computed tomography (CT) and CT angiography in all ICH patients, followed by MRI in most patients, and conventional angiography in those with a high suspicion of a secondary cause. We restricted our analysis to patients with dICH who had exclusively deep hematoma in the basal ganglia (BG), thalamus, or brainstem.^24^ Patients with multiple hematomas involving both deep and lobar territories were excluded. We also excluded patients who were <18 years, admitted more than a week after onset, those with liver cirrhosis, incomplete medical records, and poor image quality.

### Clinical assessment

We used a standardized form to collect the following information: age, sex, education, medical history including vascular risk factors (hypertension, diabetes mellitus, hyperlipidemia, coronary artery disease, chronic kidney disease, current smoking, and alcohol intake), history of stroke, pre-stroke medication (antiplatelet and lipid-lowering), and blood pressure at admission. The definition of these variables is described in Supplemental Material.

### MRI acquisition

MRI protocols included axial T1-weighted imaging (T1WI), T2-weighted imaging (T2WI), fluid-attenuated inversion recovery (FLAIR), DWI, and T2*-weighted gradient-recalled echo (GRE), or susceptibility-weighted imaging (SWI) (detailed in Supplemental Table S2). However, not all patients received GRE/SWI since they were not routine work-up in many hospitals of China.

### SVD markers

A trained neurologist (YC) blinded to clinical data and under the guidance of an expert neuroradiologist (JMW) rated SVD markers according to STRIVE criteria.^21^ Lacunes were defined as fluid-filled cavities of between 3 and 15 mm in diameter. The number of lacunes was recorded and classified topographically as lobar or non-lobar.^19^ WMH were graded using the Fazekas score in both periventricular (PVWMH) and deep (DWMH) regions, which were summed to obtain a total WMH score.^25^ Four predefined WMH topographic patterns were recorded as multiple subcortical spots, peri-basal ganglia, large anterior subcortical patches, and posterior subcortical patches.^26^ PVS were defined as <3 mm punctate or linear cerebrospinal fluid-isointense structures on T2WI. They were counted in the BG (BG-PVS) and centrum semiovale (CSO-PVS) separately and categorized as none, mild (1–10), moderate (11–20), frequent (21–40), and severe (>40) using a validated scale.^27^ CMB were defined as small (⩽10 mm) areas of signal void with associated blooming on GRE/SWI and categorized into strictly lobar or deep/mixed.^28^ Intrarater reliability testing (50 randomly selected scans) showed good reliability with kappa values of 0.92 for lacunes, 0.97 for PVWMH and DWMH, 0.96 for BG-PVS, 0.93 for CSO-PVS, and 0.95 for CMB.

### WMH segmentation and volumetric measurement

We segmented WMH using a pipeline developed by the Edinburgh SVD group in a subset of patients who had all the required sequences of adequate quality available for computational processing.^29^ Briefly, we registered all structural images to the native T2WI for LIS group, and to the native SWI for dICH group, using FSL-FLIRT (FMRIB Software Library, Oxford, UK).^30^ Next, we generated the intracranial volume (ICV) mask using a weighted average image fusion of T2WI and SWI or T2WI and FLAIR (in absence of SWI) as input to FSL-BET.^31^ Then, we identified hyperintense voxels on brain-extracted FLAIR by thresholding intensity values 1.3 times the standard deviation above the mean. Artifacts were automatically removed using a lesion distribution template derived from a study of cognitive aging.^32^ Further refinement was achieved by applying Gaussian smoothing and removing voxels with intensity values below 0.1 and *z*-scores below 0.95. We manually removed acute and old stroke lesions, and perihematomal edema on FLAIR. Final WMH masks were checked and manually corrected if necessary. WMH volume were normalized to ICV and reported as a percentage of ICV (%ICV).

### WMH probability maps and voxel-based analysis

We generated WMH probability maps using a sample of patients (*n* = 146 × 2) from both groups, matched for age, sex, and vascular risk factors. First, we conducted voxel-based comparisons of the WMH spatial distribution within each group considering individual vascular risk factors and the presence or absence of lacunes or CMB. The detailed methodology for this task was outlined in the Supplemental Material, with the software implementation adapted from our prior work (https://doi.org/10.7488/ds/3063).^33^ Then, we compared the differences in WMH spatial distribution between LIS and dICH groups. We performed voxel-based statistical comparisons using the Kruskal–Wallis test or Wilcoxon rank test, and corrected for multiple comparisons using false discovery rate. To ensure the robustness of our findings, we validated our voxel-based analyses by comparing results obtained using two different brain templates: a Caucasian age-relevant brain template, and study-specific brain templates tailored to the Chinese LIS and dICH groups. Comprehensive analysis and results were elaborated in the Supplemental Material.

### Statistical analysis

Continuous variables, expressed as mean [standard deviation (SD)] or median [interquartile range (IQR)], were analyzed using *t* test for normally distributed data, and Mann–Whitney *U* test for non-normally distributed data. Categorical variables, expressed as count (percentage), were analyzed using Chi-square or Fisher’s exact test. We assessed the demographic, clinical, and neuroimaging factors in univariable analysis stratified by stroke phenotype. Then, we assessed their association with stroke phenotype in two multivariable logistic regression models. Model 1 included age, sex, vascular risk factors, and pre-stroke medications. Model 2 included age, sex, significant variables in model one, and individual SVD markers. Normalized WMH volume were log 10 transformed before entering the model. Collinearity between predictors in all models was explored using the variance inflation factor, with a value of 5 implemented as a threshold value. Odds ratio (OR) and 95% confidence interval (CI) were reported. OR > 1 indicated respective variables more associated with dICH than LIS, while OR < 1 favored the opposite. Data were analyzed using R version 4.0.4 (R Foundation for Statistical Computing, Vienna, Austria). A two-sided *p* < 0.05 was considered statistically significant.

## Results

**Figure 1.**
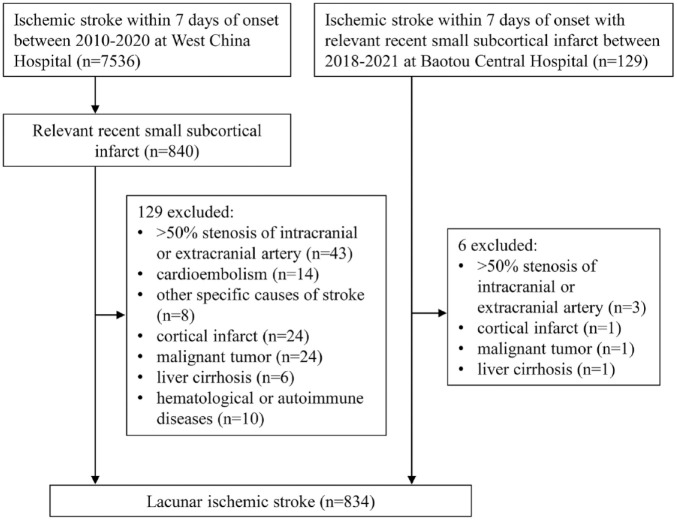
Flow diagram of patients with lacunar ischemic stroke.

**Figure 2.**
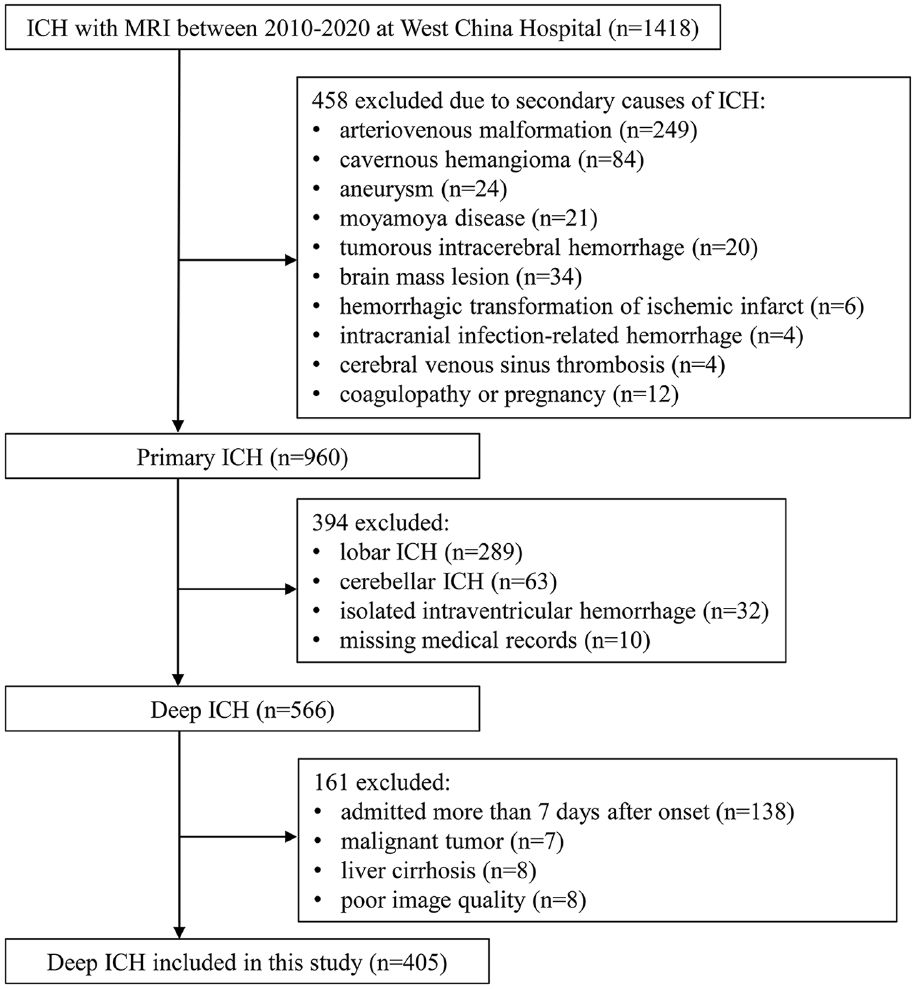
Flow diagram of patients with deep intracerebral hemorrhage.

After screening (Figures 1 and 2), we included 834 LIS patients (mean age 61.7 ± 12.1 years, 72.3% male) and 405 dICH (mean age 57.7 ± 13.2 years, 72.1% male). The median time from stroke onset to MRI scan was 4 days (IQR 2–6) for LIS, and 5 days (IQR 3–11) for dICH. For 719 LIS (86.2%) and 366 dICH patients (90.4%), the index event was the first cerebrovascular event.

**Figure 3.**
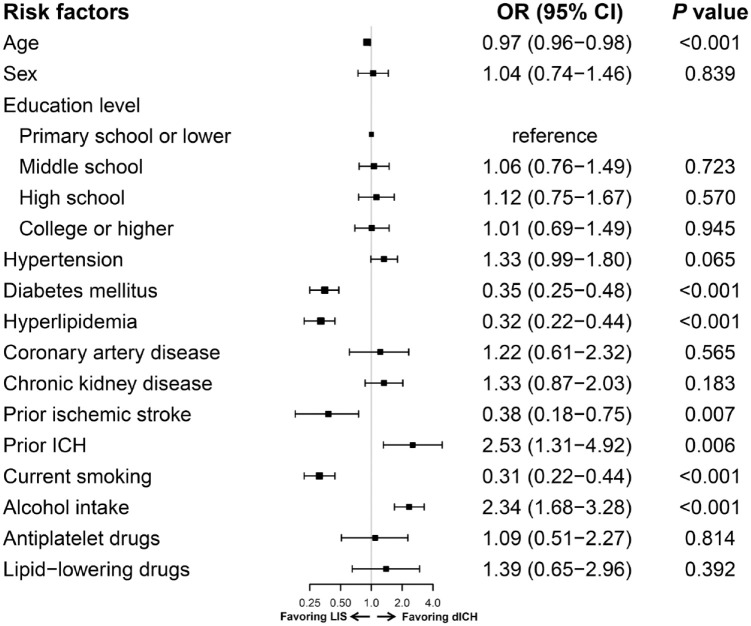
Multivariable logistic regression model for risk factors associated with dICH *versus* LIS. CI, confidence interval; dICH, deep intracerebral hemorrhage; LIS, lacunar ischemic stroke; OR, odds ratio.

The characteristics of LIS and dICH patients are shown in Table 1. Hypertension was the most prevalent vascular risk factor and was equally frequent between LIS and dICH (72.3% *versus* 74.8%, *p* = 0.349). On univariable analysis, patients with LIS were older, more frequently had diabetes mellitus, hyperlipidemia, smoking, prior ischemic stroke, and prior antiplatelet and lipid-lowering medication compared with dICH. Instead, patients with dICH more frequently had ICH history and alcohol consumption. In the multivariable model (Figure 3), diabetes mellitus (OR 0.35, 95% CI 0.25–0.48), hyperlipidemia (OR 0.32, 95% CI 0.22–0.44), current smoking (OR 0.31, 95% CI 0.22–0.44), and history of ischemic stroke (OR 0.38, 95% CI 0.18–0.75) were more significantly associated with LIS than dICH, whereas alcohol consumption (OR 2.34, 95% CI 1.68–3.28) and ICH history (OR 2.53, 95% CI 1.31–4.92) were more significantly associated with dICH.

**Table 1.** Differences in risk factor profiles between patients with LIS and dICH.

Baseline variable	LIS (*n* = 834)	dICH (*n* = 405)	*p* Value
Demographics			
Age, year, mean (SD)	61.7 (12.1)	57.7 (13.2)	<0.001
Male sex, *n* (%)	603 (72.3)	292 (72.1)	0.940
Education level, *n* (%)			0.850
Primary school or lower	294 (35.3)	134 (33.1)	
Middle school	246 (29.5)	123 (30.4)	
High school	136 (16.3)	72 (17.8)	
College or higher	158 (18.9)	76 (18.8)	
Vascular risk factors, *n* (%)			
Hypertension	603 (72.3)	303 (74.8)	0.349
Diabetes mellitus	298 (35.7)	60 (14.8)	<0.001
Hyperlipidemia	294 (35.3)	58 (14.3)	<0.001
Coronary artery disease	44 (5.3)	16 (4.0)	0.308
Chronic kidney disease	92 (11.0)	45 (11.1)	0.966
History of ischemic stroke	84 (10.1)	17 (4.2)	<0.001
History of ICH	23 (2.8)	24 (5.9)	0.006
Current smoking	346 (41.5)	124 (30.6)	<0.001
Alcohol intake	237 (28.4)	155 (38.3)	<0.001
Pre-stroke medications, *n* (%)			
Antiplatelet	101 (12.1)	29 (7.2)	0.008
Lipid-lowering	80 (9.6)	24 (5.9)	0.029
Admission blood pressure, mmHg, mean (SD)			
Systolic blood pressure	156.4 (22.9)	166.0 (27.6)	<0.001
Diastolic blood pressure	92.3 (15.2)	98.3 (16.8)	<0.001

dICH, deep intracerebral hemorrhage; LIS, lacunar ischemic stroke; SD, standard deviation.

**Figure 4.**
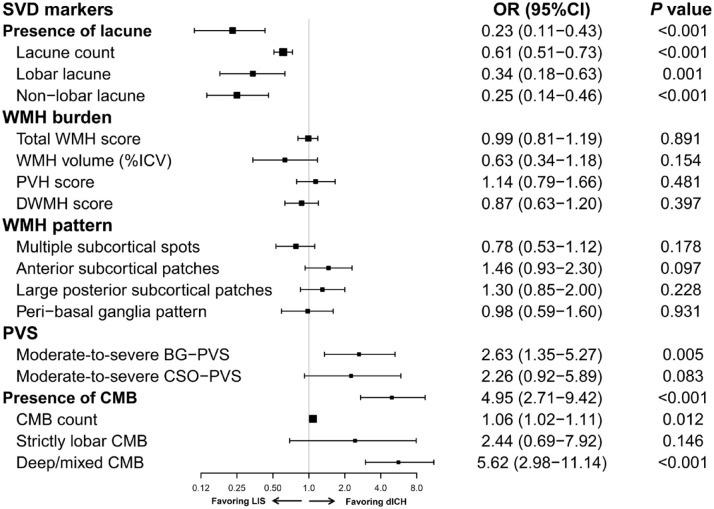
Multivariable logistic regression model for SVD markers associated with dICH *versus* LIS. Model was adjusted for age, sex, hypertension, diabetes mellitus, hyperlipidemia, prior ischemic stroke, prior intracerebral hemorrhage, current smoking, alcohol intake, presence of lacunes, total WMH score, moderate-to-severe BG-PVS, and presence of CMB. BG, basal ganglia; CI, confidence interval; CMB, cerebral microbleeds; CSO, centrum semiovale; dICH, deep intracerebral hemorrhage; ICV, intracranial volume; LIS, lacunar ischemic stroke; OR, odds ratio; PVS, perivascular spaces; SVD, small vessel disease; WMH, white matter hyperintensities.

In terms of SVD markers, lacunes were observed in 346 (41.5%) patients with LIS as compared to 143 (35.3%) patients with dICH (*p* = 0.037) (Table 2). In the multivariable model (Figure 4), presence of lacunes was more associated with LIS than dICH (OR 0.23, 95% CI 0.11–0.43) after adjustment for age, sex, hypertension, diabetes mellitus, hyperlipidemia, smoking, alcohol intake, history of ischemic stroke and ICH, and presence of other SVD markers. The association remained significant for both lobar and non-lobar lacunes (OR 0.34, 95% CI 0.18–0.63 and OR 0.25, 95% CI 0.14–0.46, respectively). Furthermore, the likelihood of having LIS increased with a higher lacune count (OR, 0.61 per 1 lacune higher count, 95% CI 0.51–0.73).

**Table 2.** Differences in MRI markers of SVD between patients with LIS and dICH.

MRI markers of SVD	LIS (*n* = 834)	dICH (*n* = 405)	*p* Value
Lacunes			
Any lacune, *n* (%)	346 (41.5)	143 (35.3)	0.037
Lacune count if lacunes present, median (IQR)	2 (1–3)	1 (1–2)	<0.001
Lobar lacune, *n* (%)	199 (23.9)	81 (20.0)	0.127
Non-lobar lacune, *n* (%)	268 (32.1)	100 (24.7)	0.007
WMH			
Total WMH score, median (IQR)	2 (1–4)	3 (1–4)	0.146
Periventricular WMH score, median (IQR)	1 (1–2)	1 (1–2)	0.141
Periventricular WMH score ⩾2, *n* (%)	354 (42.4)	187 (46.2)	0.215
Deep WMH score, median (IQR)	1 (0–2)	1 (0–2)	0.227
Deep WMH score ⩾2, *n* (%)	276 (33.1)	155 (38.3)	0.073
WMH volume, %ICV, median (IQR)^a^	0.5 (0.3–1.2)	0.5 (0.2–1.2)	0.248
WMH patterns, *n* (%)			
Multiple subcortical spots	136 (16.3)	64 (15.8)	0.821
Anterior subcortical patches	95 (11.4)	67 (16.5)	0.012
Large posterior subcortical patches	118 (14.1)	79 (19.5)	0.016
Peri-basal ganglia pattern	75 (9.0)	36 (8.9)	0.952
PVS			
BG-PVS score, median (IQR)	2 (1–2)	2 (1–2)	0.385
Moderate-to-severe BG-PVS, *n* (%)	501 (60.1)	236 (58.3)	0.545
CSO-PVS score, median (IQR)	2 (2–3)	2 (2–3)	1.000
Moderate-to-severe CSO-PVS, *n* (%)	717 (86.0)	350 (86.4)	0.830
CMB^b^			
Any CMB, *n* (%)	93/243 (38.3)	93/148 (62.8)	<0.001
CMB count if CMB present, median (IQR)	4 (2–8)	3 (2–7)	0.812
Strictly lobar CMB, *n* (%)	12/243 (4.9)	6/148 (4.1)	0.686
Deep/mixed CMB, *n* (%)	81/243 (33.3)	87/148 (58.8)	<0.001

a Available in 489 patients.

b Available in 391 patients.

BG, basal ganglia; CSO, centrum semiovale; CMB, cerebral microbleeds; dICH, deep intracerebral hemorrhage; ICV, intracranial volume; IQR, interquartile range; LIS, lacunar ischemic stroke; MRI, magnetic resonance imaging; PVS, perivascular spaces; SVD, small vessel disease; WMH, white matter hyperintensities.

The median total WMH score, PVWMH score, and DWMH score were comparable between LIS and dICH (all *p* > 0.05). WMH were segmented in a subset of 343 patients with LIS and 146 with dICH. Supplemental Table S3 shows the differences in baseline characteristics between patients with (*n* = 489) *versus* without (*n* = 750) WMH segmentation. The proportion of LIS compared to dICH did not differ between patients with and without WMH segmentation (70.1% *versus* 65.5%, *p* = 0.086). WMH volume per ICV did not differ between the two groups [LIS: median (IQR), 0.5 (0.3–1.2) *versus* dICH: 0.5 (0.2–1.2); *p* = 0.248] (Table 2). Multivariable analyses confirmed no significant association between WMH burden and stroke phenotype (OR for total WMH 0.99, 95% CI 0.81–1.19; OR for PVWMH 1.14, 95% CI 0.79–1.66; OR for DWMH 0.87, 95% CI 0.63–1.20; OR for log-transformed WMH volume 0.63, 95% CI 0.34–1.18). Regarding the topographic pattern of WMH, large anterior and posterior subcortical patches were more frequently observed in dICH compared to LIS (anterior: 16.5% *versus* 11.4%, *p* = 0.012; posterior: 19.5% *versus* 14.1%, *p* = 0.016); however, this relationship was not significant after adjustment for risk factors and WMH score (Figure 4).

The presence of moderate-to-severe BG-PVS (60.1% *versus* 58.3%, *p* = 0.545) and CSO-PVS (86.0% *versus* 86.4%, *p* = 0.83) was comparable between LIS and dICH in the univariable analysis (Table 2). However, in the multivariable analysis, moderate-to-severe BG-PVS was more significantly associated with dICH (OR 2.63, 95% CI 1.35–5.27), while CSO-PVS was not (OR 2.26, 95% CI 0.92–5.89) (Figure 4).

GRE/SWI was available to assess CMB in 243 of 834 (29.1%) patients with LIS and 148 of 405 (36.5%) patients with dICH. Baseline demographics and comorbidities were largely comparable between patients with and without GRE/SWI (Supplemental Table S4). Notably, CMB were observed in 93 (38.3%) patients with LIS compared to 93 (62.8%) patients with dICH (*p* < 0.001) (Table 2). In the multivariable analysis, presence of CMB was more significantly associated with dICH than LIS (OR 4.95, 95% CI 2.71–9.42). The association remained significant for deep/mixed CMB (OR 5.62, 95% CI 2.98–11.14) but not for strictly lobar CMB (OR 2.44, 95% CI 0.69–7.92). Moreover, the likelihood of having dICH increased with a higher CMB count (OR, 1.06 per 1 CMB higher count, 95% CI 1.02–1.11) (Figure 4).

### Voxel-based analysis

In the LIS group, the WMH distribution did not differ between patients with and without any of the assessed vascular risk factors. In the dICH group, patients with diabetes showed topological differences in 8% of the WMH voxels compared to non-diabetic patients (Supplemental Figure S1). Regarding lacunes, in the LIS group, patients with lacunes were more likely to have WMH in most white matter voxels across the brain, whereas in the dICH group, patients with lacunes were more likely to have WMH in areas surrounding the horns and superior to the lateral ventricles compared to those without lacunes. Moreover, both LIS and dICH patients with CMB were more likely to have WMH in most white matter voxels throughout the brain compared to those without CMB (Supplemental Figure S2).

**Figure 5.**
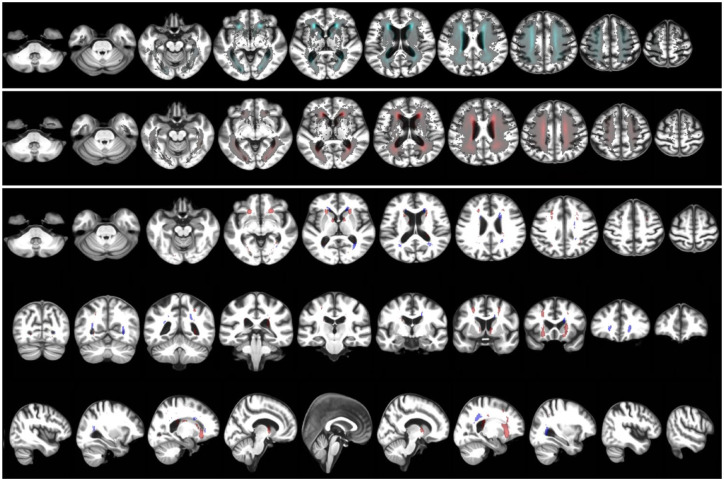
Probability distribution maps of WMH for the sample of LIS and dICH patients matched in age, sex, and vascular risk factors. The top panel is the WMH probability map of LIS sample. The middle panel is the WMH probability map of dICH sample. The bottom panel shows the differences between both maps thresholded to the highest quartile (75% or above) for LIS patients (compared to dICH) in red, and for dICH patients (compared to LIS) thresholded at 60% or above in blue, in axial, coronal, and sagittal planes. dICH, deep intracerebral hemorrhage; LIS, lacunar ischemic stroke; WMH, white matter hyperintensities.

The WMH probability maps of LIS and dICH patients from the age, sex, and vascular risk factor-matched sample were illustrated in Figure 5. The maps highlight voxels where the probability of finding a WMH in LIS and in dICH patients differed mostly, considering voxel-wise differences above a threshold of 75% for the LIS group, and above 60% for the dICH group. In direct comparison between LIS and dICH, we found a few voxels in the inferomedial white matter tracts appeared more affected in LIS than in dICH, and certain voxels in posterior thalamic radiation and some projection white matter fibers appeared more affected in dICH than in LIS, but none of these differences reached statistical significance (i.e. above 95%). All results remained consistent in the unmatched sample (details not shown). Furthermore, we validated our voxel-based analyses by comparing results obtained using two distinct brain templates: a Caucasian age-relevant brain template and a study-specific brain template, yielding similar and consistent results (Supplemental Table S5).

## Discussion

In this hospital-based study of patients presenting with clinical lacunar stroke syndrome or dICH that was confirmed on MRI, we found that LIS was more closely associated with diabetes mellitus, hyperlipidemia, history of ischemic stroke, smoking, and a higher lacune count; while dICH was more strongly associated with ICH history, alcohol consumption, moderate-to-severe BG-PVS, and a higher CMB burden. The two groups did not differ regarding the remaining cardiovascular risk factors, pre-stroke medications, WMH burden, or distribution.

A collective of 17 previous studies comparing risk factor profiles between LIS and dICH (outlined in Supplemental Table S1) yielded inconclusive results.^4–20^ These studies, with an average sample size of 346 for LIS and 198 for dICH, encompassed various study designs. Most studies (13 hospital-based, 3 population-based) enrolled LIS and dICH in the same protocol, while one study included LIS and dICH from different cohorts.^19^ Moreover, four studies exclusively involved subjects with hypertension.^8,11–13^ We extend prior research by showing hypertension was the most prevalent risk factor for LIS and dICH, without a preferential association with either condition. This finding confirms that both LIS and dICH are associated with hypertension.^2,34^ Furthermore, our study corroborates previous findings by demonstrating stronger association of diabetes mellitus,^4,8,10,15–18^ hyperlipidemia,^7–11,13,16,17^ and smoking^11–14,16–19^ with LIS compared with dICH. The differential risk factor profiles between LIS and dICH indirectly support possible distinct SVD pathology underlying the two stroke phenotypes. While lipohyalinosis and fibrinoid necrosis are both common in LIS and dICH,^35^ LIS is believed to involve mechanisms beyond intrinsic lipohyalinosis, potentially including parent or branch artery atheroma.^36^ It is plausible that the presence of the differential risk factors contributes to a more proatherogenic environment, potentially influencing LIS pathogenesis. Of note, the hypothesis largely relies on indirect assumptions from risk factor associations and post-mortem studies conducted long after acute events. However, recent Mendelian randomization research has identified positive associations of genetically determined elevated blood pressure, diabetes mellitus, and smoking with LIS, suggesting a causal role of these risk factors in LIS regardless of any relation to atheroma.^37^

We found alcohol consumption was more strongly associated with dICH than LIS, aligning with epidemiological data that alcohol intake is a more important predisposing factor for ICH than ischemic stroke.^38^ In addition, we found a history of ischemic stroke was more closely associated with LIS, while ICH history was more related to dICH. This may reflect the inherent susceptibility of ischemic or hemorrhagic brain damage to the nature of recurrent stroke and is consistent with prior studies showing that a recurrent stroke after LIS is more likely to be lacunar and after cortical large artery ischemic stroke is more likely to be cortical.^39^ Indeed, emerging evidence suggests that individuals predisposed to LIS exhibit a heightened vulnerability to develop SVD and increased susceptibility to specific risk factors.^36,40^ This emphasizes the importance of understanding not only the exposure to risk factors but how individuals respond to them.^41,42^ Similar considerations might apply to individuals predisposed to dICH. Genetic studies are needed to pinpoint the differences between LIS and dICH and potentially lead to more targeted treatments.

Few studies have investigated the relation of SVD markers to the stroke phenotype.^42,43^ Our findings are consistent with a previous study by Wiegertjes *et al*.,^19^ encompassing 82 LIS and 54 dICH patients, which showed a preferential association of lacunes with LIS and CMB with dICH. It should be noted, however, their study had certain limitations, including a relatively small sample size and the inclusion of a heterogeneous population from two distinct cohorts with variability in risk factor definitions. Furthermore, their retrospective selection of LIS patients from a radiological SVD cohort may have led to imprecise estimates of association since all participants already exhibited certain SVD markers (WMH or lacunes) at inclusion. In contrast, our study collected a large sample from a hospital-based stroke registry, ensuring clinically evident stroke diagnosis and subtyping, and consistent definitions of comorbidities. However, our findings differ from observations from a recent Swiss study involving 599 LIS and 117 dICH patients, which reported an unadjusted association of lacunes with dICH over LIS.^20^ The discrepancy partly relates to the notably higher prevalence of lacunes in the dICH group reported in the Swiss study (60.5%) compared with previous studies (around 20–30%).^44^ Taken together, lacunes and CMB may be useful in distinguishing risk for ischemic and hemorrhagic SVD, possibly preceding clinical events, although more evidence from longitudinal studies is needed to determine this.

Regarding WMH, neither our study nor Wiegertjes *et al*.^19^ showed an association of WMH burden or distribution pattern with any stroke phenotype. One study involving 352 hypertensive patients with LIS and 219 dICH reported that PVWMH score predicted LIS over dICH.^13^ Another two studies (*n* = 172 and 286, respectively) found that CT-rated white matter lesions favored LIS over dICH.^5,18^ However, these disparities could arise from differences in study design and population characteristics. The pathogenesis of WMH remains poorly defined, as conventional vascular risk factors explain only ~2% of variance in WMH.^41^ In our analysis, we did not observe voxel-wise differences in WMH spatial distribution in relation to any vascular risk factor, except for an 8% topological difference in WMH voxels between diabetic and non-diabetic in the dICH group. Moreover, the presence of lacunes or CMB in both LIS and dICH patients heightened the likelihood of widespread WMH presentation, suggesting an increased susceptibility to accumulating SVD with minimal impact from vascular risk factors. Together with the limited effect of vascular risk factor interventions (e.g. blood-pressure lowering) on WMH or LIS,^45,46^ this indicates a large unexplained variance due to other factors, including genetic predisposition, in the biologic relationship between WMH occurrence and stroke phenotypes.

Our study provided a novel evidence that moderate-to-severe BG-PVS was more strongly associated with dICH than LIS. Although the proportion of moderate-to-severe BG-PVS was similar between the two groups in univariable analysis, it became a significant predictor of dICH in multivariable modeling. This might be explained by the fact that many LIS patients with moderate-to-severe BG-PVS had more coexisting lacunes than those with none-to-mild BG-PVS (52.7% *versus* 24.6%). After adjusting for lacunes, evident reverse confounding was observed, demonstrating that BG-PVS was in fact a predisposing factor for dICH. Indeed, several studies have shown independent associations between higher BG-PVS burden and incident ICH among community-dwelling individuals and ischemic stroke patients.^47–49^ The association between BG-PVS and dICH might be mediated by a shared underlying mechanism. Arterial stiffness is linked to both BG-PVS and dICH.^50,51^ This condition is thought to reflect reduced damping of the cardiac impulse and thus potentially enhanced transmission of pulsatile force to small perforating arteries. Consequently, arterial stiffening might increase the risk of dICH and promote PVS formation by affecting small vessel pulsatility, which is thought to be a driver of perivascular fluid transport.^52^ If validated in prospective studies with computational analysis of PVS count and volume, BG-PVS might serve as an early marker to predict the risk of hemorrhagic SVD.

Strengths of our study include large patient sample, detailed stroke phenotyping using MRI, and comprehensive assessment of SVD markers with validated rating scales and quantitative measure. Our study has some limitations. First, this is a retrospective analysis of data from a stroke registry, so hospital referral bias might exist. Second, not all patients underwent MRI, particularly those with severe ICH, possibly introducing selection bias. Besides, two-thirds of our sample lacked GRE/SWI sequence, precluding CMB assessment. Nonetheless, baseline demographics and comorbidities were comparable between patients with and without GRE/SWI. Third, variations in the field strengths of MRI scanners and sequence parameters between the two centers might impact the detection of SVD markers, including WMH volume and voxel-based analysis. However, our utilization of both qualitative and quantitative methods yielded consistent findings. Fourth, we did not collect information on the severity, duration, and management of comorbidities, potentially leading to residual confounding. On the other hand, the risk factor profile investigated in our study only included conventional cardiovascular risk factors. Future research should explore the influence of additional factors, including genetics, inflammation, and environmental influences. Fifth, since the study was conducted in China, the generalizability of the findings to other populations may be limited. Lastly, given its cross-sectional design, our study implied association rather than causation. Further prospective studies across diverse populations are imperative to better establish the relative impact of risk factors, emerging SVD features and their progression on the occurrence of different stroke phenotype.

## Conclusion

In summary, LIS and dICH are both considered as consequences of deep perforator arteriopathy, but they present distinct risk factor profiles and predominant SVD markers. Further longitudinal studies with advanced imaging, pathology, and genetics are needed to clarify the pathophysiologic mechanisms behind the development of specific stroke phenotypes and to develop mechanism-based treatments.

## Supplemental Material

sj-docx-1-tan-10.1177_17562864241253901 – Supplemental material for Differential risk factor profile and neuroimaging markers of small vessel disease between lacunar ischemic stroke and deep intracerebral hemorrhage Supplemental material, sj-docx-1-tan-10.1177_17562864241253901 for Differential risk factor profile and neuroimaging markers of small vessel disease between lacunar ischemic stroke and deep intracerebral hemorrhage by Yajun Cheng, Maria del C. Valdés Hernández, Mangmang Xu, Shuting Zhang, Xiaohua Pan, Baoqiang An, Joanna M. Wardlaw, Ming Liu and Bo Wu in Therapeutic Advances in Neurological Disorders

sj-docx-2-tan-10.1177_17562864241253901 – Supplemental material for Differential risk factor profile and neuroimaging markers of small vessel disease between lacunar ischemic stroke and deep intracerebral hemorrhage Supplemental material, sj-docx-2-tan-10.1177_17562864241253901 for Differential risk factor profile and neuroimaging markers of small vessel disease between lacunar ischemic stroke and deep intracerebral hemorrhage by Yajun Cheng, Maria del C. Valdés Hernández, Mangmang Xu, Shuting Zhang, Xiaohua Pan, Baoqiang An, Joanna M. Wardlaw, Ming Liu and Bo Wu in Therapeutic Advances in Neurological Disorders

## Declarations

**Ethics approval and consent to participate:** This study complies with the principles of the Declaration of Helsinki. The study was approved by the Biomedical Research Ethics Committee of West China Hospital (2016 [335]) and Ethics Committee of Baotou Central Hospital (2023-WZ-029). The need for written informed consent was waived given the retrospective nature of the study.

**Consent for publication:** Not applicable.

**Availability of data and materials:** Anonymized data are available upon reasonable request from qualified investigators. Voxel-based analyses processing code, cohort brain templates, and probabilistic image maps will be publicly available from Edinburgh DataShare. The code for segmenting ICV and WMH is publicly available from https://datashare.ed.ac.uk/handle/10283/8501.
