# Supplementary material for: Differential risk factor profile and neuroimaging markers of small vessel disease between lacunar ischemic stroke and deep intracerebral hemorrhage
Source: Ther Adv Neurol Disord. 2024 May 23;17:17562864241253901. doi: 10.1177/17562864241253901 (PMC11119384; doi:10.1177/17562864241253901)
Supplement: sj-docx-1-tan-10.1177_17562864241253901 – Supplemental material for Differential risk factor profile and neuroimaging markers of small vessel disease between lacunar ischemic stroke and deep intracerebral hemorrhage [file sj-docx-1-tan-10.1177_17562864241253901.docx]

**SUPPLEMENTARY MATERIAL**

**Table S1.** Overview of studies on the comparison of risk factors and imaging features between LIS and deep ICH.

| **Study** | **Country** | **Setting** | **Sample size** | **Imaging** | **LIS and ICH definition** | **Mean age (SD)** | **Risk factors assessed** | **SVD markers assessed** | **Associations with LIS versus ICH** |
| --- | --- | --- | --- | --- | --- | --- | --- | --- | --- |
| van Zagten, 1994^1^ | Netherlands | Hospital-based registry; LIS and ICH were enrolled in the same protocol. | LIS (n=242), ICH (n=83) | CT | **LIS:** lacunar syndrome without relevant lesion; or <20 mm subcortical infarct on CT.  **ICH:** primary ICH in basal ganglia. | LIS: 68 ICH: 68 | Age, sex, hypertension, DM, ischemic heart disease, cardiac embolism. | No | Multivariable logistic regression analysis showed that DM and ischemic heart disease favored LIS. |
| Janssens, 1995^2^ | France | Hospital-based study; LIS and ICH were enrolled in the same protocol. | LIS (n=108), ICH (n=64) | CT | **LIS:** <15 mm subcortical infarct, most of them being lacunes.  **ICH:** primary ICH involving the basal ganglia, thalamus, internal capsule, cerebellum or brain stem and sparing the cortical surface. | LIS: median 71 (range 26-94) ICH: 61 (39-85) | Age, sex, hypertension, DM, hyperlipemia, MI, current drinking, migraine, AF, valvulopathy, left ventricular hypertrophy, ICA stenosis of 50% or more. | leukoaraiosis score on CT | Stepwise logistic regression analysis showed higher leukoaraiosis score favored LIS; hypertension, alcoholism, and lack of significant ICA stenosis favored ICH. |
| Schmal, 1998^3^ | Italy | Population-based stroke registry. LIS and ICH were enrolled in the same protocol. | LIS (n=242), ICH (n=68) | CT or MRI | **LIS:** lacunar syndrome without relevant lesion; or <20 mm subcortical infarct on CT or MRI. Patients with cardiac embolic source were excluded.  **ICH:** primary ICH in basal ganglia/internal capsule. | LIS: 74 (11) ICH: 71 (12) | Age, sex, hypertension, DM, smoking, hypercholesterolemia, family history of vascular events, peripheral arterial disease, ischemic heart disease, carotid stenosis > 50%. | No | Multivariable logistic regression analysis showed that younger age favored ICH. |
| Beltrán, 1998^4^ | Spain | NA | LIS (n=204), ICH (n=163) | CT | **LIS:** first-ever lacunar infarcts.  **ICH:** deep ICH. | NA | TIA, hypertension, DM, hypercholesterolemia, ischemic cardiopathy, AF. | Silent infarcts on CT | Multivariable logistic regression analysis showed that TIA, hypercholesterolemia and the presence of silent lacunar-type infarcts on CT favored LIS; arterial blood pressure favored ICH. |
| Tsivgoulis, 2005^5^ | Greece | Hospital-based registry; LIS and ICH were enrolled in the same protocol. | LIS (n=92), ICH (n=67), both with history of hypertension | CT or MRI | **LIS:** lacunar syndrome with a subcortical or brainstem lesion (CT/MRI) of <15 mm. Patients with cardiac embolic source and large vessel atherosclerotic changes were excluded.  **ICH:** Primary ICH in lobar, lenticulocapsular, thalamic, cerebellar or brainstem. | LIS: 65 (9.6) ICH: 64.9 (10.6) | Age, sex, BMI, duration of hypertension, DM, hypercholesterolemia, smoking, alcohol intake, CAD, TIA, current antihypertensive, blood sugar- and lipid-lowering medications, CCA-IMT. | No | Multivariable logistic regression analysis showed that CCA-IMT, DM and hypercholesterolemia favored LIS. |
| Ronquillo, 2007^6^ | Cuba | Hospital-based study; LIS and ICH were enrolled in the same protocol. | LIS (n=46), ICH (n=39) | CT | **LIS:** clinical diagnosis corroborated by CT.  **ICH:** clinical diagnosis corroborated by CT. | LIS: 68.5 ICH: 70.9 | Age, sex, hypertension, DM, hyperlipidemia, smoking, previous TIA/ischemic stroke, ischemic heart disease, AF. | No | Univariate comparison analysis showed that hyperlipidemia favored LIS. |
| Labovitz, 2007^7^ | US | Population-based study; LIS and ICH were enrolled in the same protocol. | LIS (n=151), ICH (n=83) | CT | **LIS:** lacunar syndrome without relevant lesion; or <15 mm infarct in the appropriate territory visible on CT. Patients with cardiac embolus or significant extracranial or intracranial artery stenosis were excluded.  **ICH:** primary ICH in the basal ganglia, thalamus, cerebellum, or pon. ICH associated with warfarin or other hypocoagulable states was not excluded. | LIS: 67 (range 29–92) ICH: 62 (range 34–98) | Hypertension, DM, current smoking, alcohol abuse, drug abuse, cholesterol level. | No | Multivariable logistic regression analysis showed that older age, DM, and higher cholesterol levels favored LIS. |
| Cortina, 2008^8^ | Spain | Hospital-based stroke registry; LIS and ICH were enrolled in the same protocol. | LIS (n=129), ICH (n=107), both with history of hypertension | CT | **LIS:** TOAST criteria. Embolic heart disease and carotid atherosclerosis were excluded.  **ICH:** primary ICH involving predominately the basal ganglia, periventricular white matter or internal capsule. | LIS: 69.8 (12.8) ICH: 71.7 (11.9) | Age, sex, DM, hyperlipidemia, current smoking, alcohol overuse, blood biomarkers. | No | Multivariable logistic regression analysis showed that current smoking, hyperlipidemia, higher monocyte counts favored LIS; lower cholesterol, triglyceride and higher neutrophil counts favored ICH. |
| Kaplan, 2014^9^ | US | Hospital-based study; LIS and ICH were enrolled in the same protocol. | LIS (n=363), ICH (n=217), both with history of hypertension | CT or MRI | **LIS:** a single, focal, MRI visible lesion <20 mm in subcortical structures. Patients with cardioembolic source were excluded.  **ICH:** hemorrhage in the subcortical white matter or deep structures. | LIS: 68 ICH: 68 | Age, race, sex, smoking, alcohol, drug abuse. | No | Multivariable analysis showed that smoking favored LIS; drug abuse appeared to favor ICH. |
| Marsh. 2014^10^ | US | Hospital-based study; LIS and ICH were enrolled in the same protocol. | LIS (n=352), ICH (n=219), both with history of hypertension | CT or MRI | **LIS:** a focal lesion measuring <20 mm in subcortical structures visible on MRI or CT that corresponded to the stroke symptoms. Patients with cardioembolic source were excluded.  **ICH:** subcortical ICH. | LIS: 66.3 (14) ICH: 65.0 (14) | Age, race, sex, SBP, DBP, DM, left ventricular hypertrophy, alcohol or tobacco use; serum creatinine, glucose, hematocrit, platelet count, AST, ESR, INR, HDL, LDL. | Periventricular WMH score, cerebral microbleeds on MRI | Multivariable logistic regression analysis showed that higher levels of LDL and periventricular WMH grade favored LIS; higher levels of ESR, AST, INR, and presence of cerebral microbleeds favored ICH. |
| Morotti, 2016^11^ | Italy | Hospital-based registry. LIS and ICH were enrolled in the same protocol. | LIS (n=1434), ICH (n=497) | CT | **LIS:** lacunar syndrome. Patients with cardioembolic source or ipsilateral carotid artery disease were excluded.  **ICH:** primary ICH in basal ganglia, thalamus, or brainstem. ICH associated with oral anticoagulants was not excluded. | LIS: 71.1 (13.3) ICH: 71.7 (13.2) | Age, sex, hypertension, DM, hypercholesterolemia, CAD, current smoking, excessive alcohol intake, SBP, DBP, previous use of statin, antiplatelet and anticoagulant therapy. | No | Multivariable logistic regression analysis showed that current smoking favored LIS; hypertension, excessive alcohol use, anticoagulant, and statin therapy favored ICH. |
| Lioutas, 2017^12^ | US | Nested case–control study within the Framingham Heart Study comparing people with incident LIS and ICH. | 118 LIS (n=118), ICH (n=106) | NA | **LIS:** lacunar syndrome with imaging to confirm.  **ICH:** primary ICH involving the basal ganglia, thalamus, corona radiata or brainstem and sparing the cortex. | LIS: 74 (10) ICH: 75 (13) | Age, sex, education, BMI, SBP, DBP, hypertension, DM, cardiovascular disease, AF, smoking, total cholesterol, HDL, anticoagulant use. | No | Multivariable logistic regression analysis showed that DM and higher BMI favored LIS. |
| Chen, 2019^13^ | China | Hospital-based stroke registry. LIS and ICH were enrolled in the same protocol. | LIS (n=1135), ICH (n=1125) | CT or MRI | **LIS:** lacunar syndrome and with a relevant subcortical infarct (documented by CT/MRI) with a diameter <15 mm. Patients with cardiac embolic source and ipsilateral large extracranial arteries showing a stenosis of >50% were excluded.  **ICH:** primary ICH in the putamen, caudate nucleus, internal capsule, thalamus, or brainstem. | LIS: 64.2 (11.8) ICH: 61.7 (12.3) | Age, sex, hypertension, SBP, DBP, DM, CAD dyslipidemia, current smoking, alcohol consumption, BMI, waist–height ratio, WBC, platelet, eGFR, lipid profiles. | No | Multivariable logistic regression analysis showed that male sex, DM, smoking, higher atherogenic lipid profiles, BMI, waist–height ratio, platelet and abnormal eGFR favored LIS; higher blood pressure, WBC, hs-CRP, and HDL favored ICH. |
| Bernal, 2021^14^ | Spain | Hospital-based stroke registry. LIS and ICH were enrolled in the same protocol. | LIS (n=440), ICH (n=210) | CT and/or MRI | **LIS:** lacunar syndrome and CT/MRI were either normal or demonstrated only small, local brain lesions with diameter <20 mm located in perforating vessel territory. Patients with supra-aortic arterial stenosis >50% or cardioembolism were excluded.  **ICH:** primary ICH in subcortical areas including internal capsule, basal ganglia, and thalamus. | LIS: 73.5 (10.4) ICH: 73.5 (11.8) | Age, sex, hypertension, cardiac diseases, DM, chronic liver disease, dyslipidemia, history of cerebrovascular disease, TIA, current smoking, alcohol use, anticoagulant therapy. | No | Multivariable logistic regression analysis showed that DM, dyslipidemia, and current smoking favored LIS; anticoagulant therapy and chronic liver disease favored ICH. |
| Muscari, 2021^15^ | Italy | Hospital-based study. LIS and ICH were enrolled in the same protocol. | LIS (n=143), ICH (n=143) | CT | **LIS:** ischemic lesion <15 mm in deep location (internal capsule, basal ganglia, thalamus, corona radiata, brainstem), with lacunar symptoms or posterior circulation symptoms. Patients with AF and relative anticoagulant therapy were excluded.  **ICH:** deep ICH. | LIS: 70 (13) ICH: 71.6 (12.6) | Age, sex, BMI, hypertension, DM, hypercholesterolemia, hypertriglyceridemia, smoking, AF prior ischemic or hemorrhagic stroke, myocardial infarction, peripheral artery disease, chronic kidney disease, antiplatelet drug, anticoagulant drug, statin, routine laboratory data, ECG and echocardiographic parameters. | White matter lesions on CT | Multivariable logistic regression analysis showed that ever smoking, diabetes, white matter lesions favored LIS; average left ventricular wall thickness and AF favored ICH. |
| Wiegertjes, 2021^16^ | Netherlands | Two prospective cohort studies; LIS and ICH were enrolled with different protocol. | LIS (n=82), ICH (n=54) | MRI | **LIS:** lacunar stroke/TIA with subcortical MRI lesions, patients with other causes of stroke were excluded.  **ICH:** primary ICH in the basal ganglia, thalamus, brainstem or cerebellum. | LIS: median 63 [57–72] ICH: median 66 [59–75] | Age, sex, hypertension, DM, smoking, alcohol overuse, BMI. | WMH volume, lacunes, cerebral microbleeds on MRI | Multivariable logistic regression analysis showed that male sex, hypertension, smoking, presence of lacunes favored LIS; presence of cerebral microbleeds favored ICH. |
| Goeldlin, 2023^17^ | Switzerland | Hospital-based stroke registry. LIS and ICH were enrolled in the same protocol. | LIS (n=599), ICH (n=117) | MRI | **LIS:** DWI lesion in MRI or CT perfusion deficit in acute imaging plus follow-up DWI and/or FLAIR lesion in the same localization and sudden-onset corresponding clinic. LIS with non-deep perforator arteriolopathy etiology were excluded.  **ICH:** primary ICH in the basal ganglia, thalamus, brainstem. Other etiologies were excluded. | LIS: 69.7 (13.6) ICH: 65.1 (15.2) | Age, sex, hypertension, DM, hyperlipidemia, AF, history of stroke, previous use of antihypertensives, antiplatelet, anticoagulant, lipid-lowering therapy. | WMH, lacunes, perivascular spaces, cerebral microbleeds, SVD score on MRI | In univariable analysis, lacunes and cerebral microbleeds were more prevalent in ICH, while basal ganglia perivascular spaces were more prevalent in LIS. Multivariable analysis showed that higher SVD burden score favored ICH. |

LIS: lacunar ischemic stroke; ICH: intracerebral hemorrhage; SD: standard deviation; SVD: small vessel disease; CT: computed tomography; MRI: magnetic resonance imaging; DM: diabetes mellitus; MI: myocardial infarction; AF: atrial fibrillation; ICA: internal carotid artery; TIA: transient ischemic attack; BMI: body mass index; CAD: coronary artery disease; CCA-IMT: intima-media thickness of the common carotid artery; TOAST: Trial of Org 10172 in Acute Stroke Treatment; SBP: Systolic blood pressure; DBP: Diastolic blood pressure; AST: aspartate transaminase; ESR: erythrocyte sedimentation rate; HDL: high-density lipoprotein; INR: international normalized ratio; LDL: low-density lipoprotein; WMH: white matter hyperintensity; WBC: white blood cell; PLT: platelet; eGFR, estimated glomerular filtration rate; ECG: electrocardiogram; DWI: diffusion-weighted imaging; FLAIR: fluid-attenuated inversion recovery; NA: not available.

**Supplemental Methods**

*Risk factor definition*

Hypertension was defined as systolic blood pressure ≥140 mmHg, diastolic blood pressure ≥90 mmHg out of the acute phase, or treatment with antihypertensive drugs. Diabetes mellitus was defined as at least two random glucose measurements ≥11.1 mmol/L, fasting blood glucose measurements ≥7.0 mmol/L, glycosylated hemoglobin A1c ≥6.5%, or treatment with antidiabetic drugs. Hyperlipidemia was defined as a fasting triglyceride level >1.7 mmol/L, total cholesterol >5.7 mmol/L, or use of lipid-lowering drugs. Chronic kidney disease was defined as an estimated glomerular filtration rate <60 mL/min/1.73m^2^. Current smoking was defined as consuming at least one cigarette per day or quit smoking ≤1 year. Alcohol intake was defined as consuming >50 g alcohol per day. The definition of coronary artery disease and stroke history was based on the International Classification of Diseases, 9th version.

*MRI protocol*

**Table S2.** MRI sequence parameters for each of the scanners where patients had their brain MRI acquired.

| **Hospital** | **MRI scanner** | **Sequence** | **TR/TE/TI (ms)** | **Voxel size (mm)** | **Slices** |
| --- | --- | --- | --- | --- | --- |
| West China Hospital | 3 T TrioTim, Siemens | DWI | 4000/91 | 0.57 x 0.57 x 5 | 21 |
|  |  | T1WI | 1600/9.2 | 0.69 x 0.69 x 5 | 21 |
|  |  | T2WI | 4000/93 | 0.34 x 0.34 x 5 | 21 |
|  |  | FLAIR | 9000/93/2498.2 | 0.43 x 0.43 x 5 | 21 |
|  |  | SWI | 28/20 | 0.6 x 0.6 x 2 | 72 |
| West China Hospital | 3 T Skyra, Siemens | DWI | 6400/98 | 0.57 x 0.57 x 5 | 21 |
|  |  | T1WI | 1600/8.6 | 0.9 x 0.9 x 5 | 21 |
|  |  | T2WI | 4250/98 | 0.69 x 0.69 x 5 | 21 |
|  |  | FLAIR | 8500/85/2439 | 0.47 x 0.47 x 5 | 21 |
|  |  | SWI | 27/20 | 0.9 x 0.9 x 2 | 64 |
| West China Hospital | 3 T Achieva, Philips | DWI | 1528/47 | 0.9 x 0.9 x 6 | 18 |
|  |  | T1WI | 2000/10 | 0.45 x 0.45 x 6 | 18 |
|  |  | T2WI | 3000/80 | 0.45 x 0.45 x 6 | 18 |
|  |  | FLAIR | 11000/120/2800 | 0.45 x 0.45 x 6 | 18 |
| West China Hospital | 3 T DISCOVERY_MR750w, G.E. | DWI | 4880/77.7 | 0.94 x 0.94 x 5 | 20 |
|  |  | T1WI | 2470/21 | 0.47 x 0.47 x 5 | 20 |
|  |  | T2WI | 5468/125 | 0.47 x 0.47 x 5 | 20 |
|  |  | FLAIR | 9000/96/2474 | 0.47 x 0.47 x 5 | 20 |
|  |  | SWI | 37/22 | 0.47 x 0.47 x 2.5 | 52 |
| West China Hospital | 1.5 T Avanto, Siemens | DWI | 3000/87 | 1.8 x 1.8 x 5.5 | 19 |
|  |  | T1WI | 390/8.4 | 0.45 x 0.45 x 5.5 | 19 |
|  |  | T2WI | 4100/93 | 0.72 x 0.72 x 5.5 | 19 |
|  |  | FLAIR | 6000/89/2030 | 0.45 x 0.45 x 5.5 | 19 |
| Baotou Central Hospital | 1.5 T Avanto, Siemens | DWI | 3000/89 | 1.2 x 1.2 x 5 | 19 |
|  |  | T1WI | 450/11 | 0.45 x 0.45 x 5 | 19 |
|  |  | T2WI | 5300/110 | 0.72 x 0.72 x 5 | 19 |
|  |  | FLAIR | 9000/109/2500 | 0.45 x 0.45 x 5 | 19 |
|  |  | GRE | 800/26 | 0.45 x 0.45 x 5 | 19 |
| Baotou Central Hospital | 3 T Verio, Siemens | DWI | 6100/86 | 1.2 x 1.2 x 5 | 19 |
|  |  | T1WI | 220/2.5 | 0.72 x 0.72 x 5 | 19 |
|  |  | T2WI | 4000/114 | 0.6 x 0.6 x 5 | 19 |
|  |  | FLAIR | 8600/94/2450 | 0.45 x 0.45 x 5 | 19 |
|  |  | SWI | 27/20 | 0.98 x 0.98 x 2 | 56 |

MRI: magnetic resonance imaging. TR: repetition time; TE: echo time; TI: inversion time; DWI: diffusion weighted imaging; FLAIR: fluid-attenuated inversion recovery; SWI: susceptibility weighted image; GRE: gradient recalled echo.

*Voxel-based analyses*

Individual intracranial volume (ICV) masks were semi-rigidly aligned to a standard Caucasian age-relevant brain template^18^ for voxel-based analyses comparing dICH and LIS patient groups.^19^ The transformations were applied to the white matter hyperintensity (WMH) masks, which, in this way, were semi-rigidly aligned in the template space. These semi-rigid alignments were performed using ‘niftyreg’^20^ through tractor.^21^ 4D arrays comprised of the aligned WMH masks from each patient group were constructed, to compare patient groups attending to each vascular risk factor, and presence or not of lacunes or cerebral microbleeds (CMB), adapting the software implementation in Valdés Hernández et al.^22^ The voxel-based statistical comparisons used the Kruskal-Wallis test when more than two subgroups were compared (e.g. alcohol consumption level and smoking status), and the Wilcoxon rank test when comparing two subgroups (i.e. having the vascular risk factor or not, or LIS vs. dICH). We used false discovery rate (FDR) for correcting our voxel-based results for multiple comparisons.

**Figure S1.** Voxels where the WMH distribution differed between diabetics and non-diabetics in the dICH group, after FDR correction at *P*<0.01 (yellow areas) and *P*<0.05 (red areas).


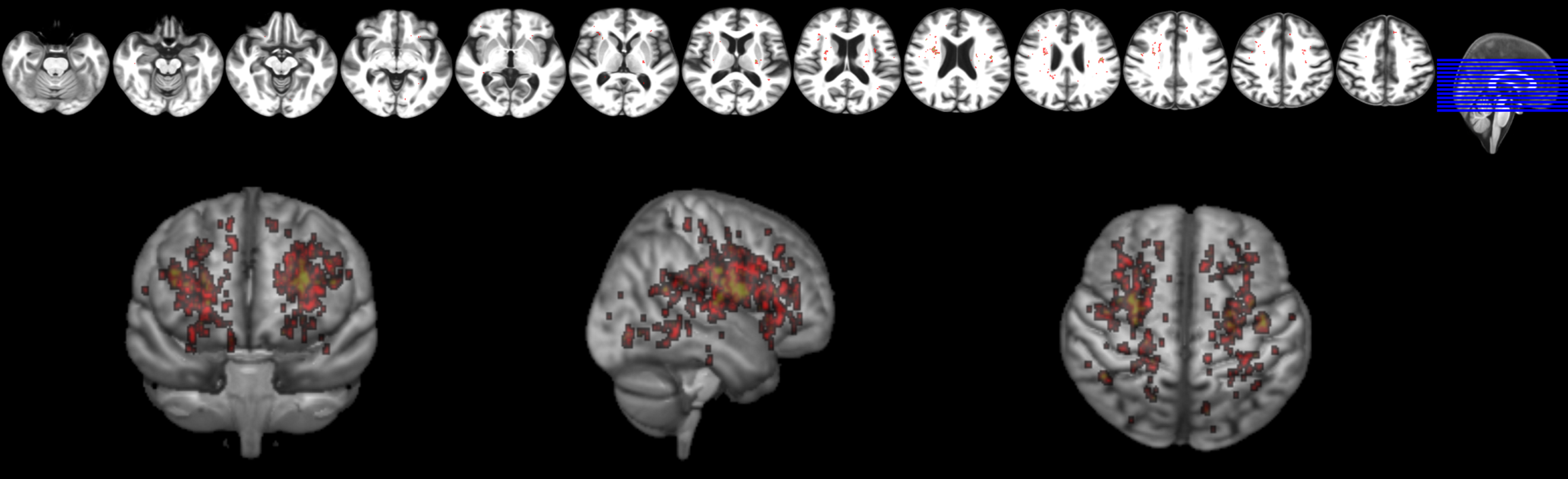


**Figure S2.** Voxels where the WMH distribution differed between patients that had lacunes vs. those who did not have (top row in both panels) and patients who had CMB vs. those who did not have (bottom row in both panels) in the LIS group (panel above) and dICH group (panel below) after FDR correction at *P*<0.01 (yellow areas) and *P*<0.05 (red areas).


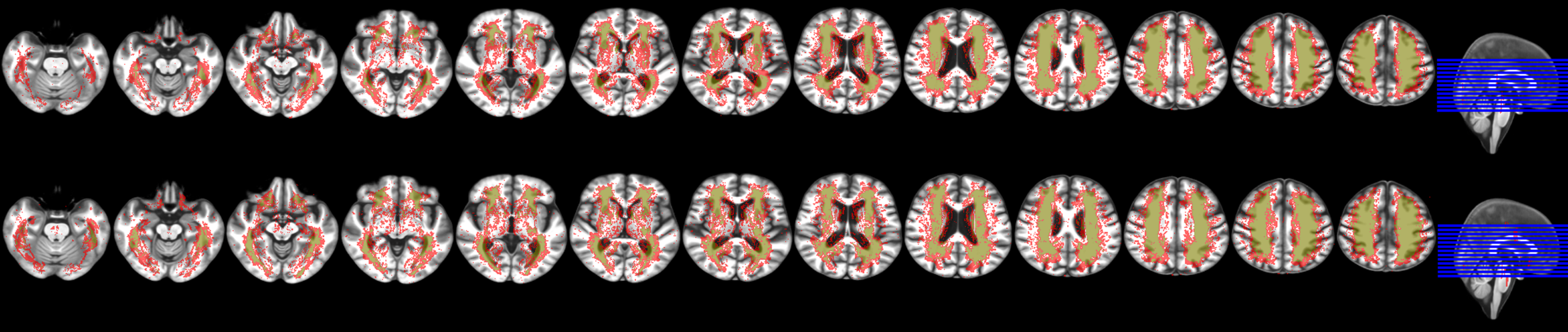


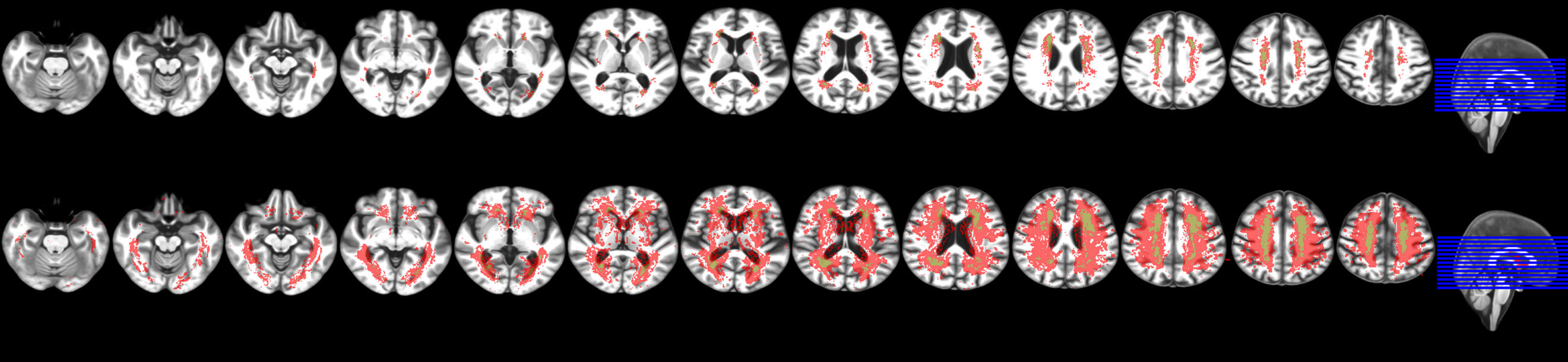


*Validation of the results from the voxel-based analyses*

Given known morphological differences between Caucasian and Chinese populations,^23^ we generated a study-specific template for each patient group, repeated the voxel-based analyses using the study-specific templates, and calculated the differences in the results obtained from aligning the WMH binary masks to each template at a voxel level. To construct the study-specific templates we selected representative brains from each patient group (LIS or dICH) using the Mahalanobis distance.^24^ This was calculated considering WMH volume, ICV and percentage of brain tissue volume in ICV. The representative brains form each patient group were rigidly aligned to the Caucasian age-relevant brain template without resampling, using FSL-FLIRT.^25^ Then, the process of semi-rigidly aligning the WMH masks to each template was followed as described previously. **Table S5** shows the number of voxels of the WMH probability distribution maps statistically significantly different in each patient group between patients in terms of vascular risk factors and presence of lacunes or CMB, from the analyses considering each template, after correcting for multiple comparisons. As can be appreciated, similar results were obtained regardless of the brain template used. The differences in the number of voxels with statistically significant results using one template over the other were in the range from 0 to 2.18% of the total number of voxels different from zero in the respective WMH probability distribution maps. Spatial distribution of the voxels showing significant group differences were consistent using both types of templates.

**Table S3.** Comparison between patients with or without WMH segmentation.

|  | **WMH segmentation (n=489)** | **No WMH segmentation (n=750)** | ***P* value** |
| --- | --- | --- | --- |
| **Demographics** |  |  |  |
| Age, year, mean (SD) | 60.2 (12) | 60.5 (12.9) | 0.637 |
| Male sex, n (%) | 361 (73.8) | 534 (71.2) | 0.313 |
| **Stroke type, n (%)** |  |  | 0.086 |
| LIS | 343 (70.1) | 491 (65.5) |  |
| dICH | 146 (29.9) | 259 (34.5) |  |
| **Vascular risk factors, n (%)** |  |  |  |
| Hypertension | 365 (74.6) | 541 (72.1) | 0.330 |
| Diabetes mellitus | 148 (30.3) | 210 (28) | 0.390 |
| Hyperlipidemia | 118 (24.1) | 234 (31.2) | 0.007 |
| Coronary artery disease | 31 (6.3) | 29 (2.9) | 0.047 |
| Chronic kidney disease | 43 (8.8) | 94 (12.5) | 0.040 |
| Prior ischemic stroke | 51 (10.4) | 50 (6.7) | 0.018 |
| Prior ICH | 19 (3.9) | 28 (3.7) | 0.891 |
| Current smoking | 194 (39.7) | 276 (36.8) | 0.308 |
| Alcohol intake | 143 (29.2) | 249 (33.2) | 0.143 |
| **Pre-stroke medications, n (%)** |  |  |  |
| Antiplatelet | 56 (11.5) | 74 (9.9) | 0.373 |
| Lipid-lowering | 46 (9.4) | 58 (7.7) | 0.299 |
| **Admission blood pressure, mmHg,** **mean (SD)** |  |  |  |
| Systolic blood pressure | 160.7 (25.6) | 158.8 (24.5) | 0.193 |
| Diastolic blood pressure | 95.1 (16.1) | 93.7 (15.9) | 0.127 |
| **MRI markers of SVD** |  |  |  |
| Presence of lacune, n (%) | 253 (51.7) | 236 (31.5) | <0.001 |
| Total WMH score, median [IQR] | 3 [2–4] | 2 [1–4] | 0.072 |
| BG-PVS score, median [IQR] | 2 [1–2] | 2 [1–2] | <0.001 |
| Moderate-to-severe BG-PVS, n (%) | 313 (64) | 424 (56.5) | 0.009 |
| CSO-PVS score, median [IQR] | 2 [2–3] | 2 [2–3] | 0.362 |
| Moderate-to-severe CSO-PVS, n (%) | 433 (88.5) | 634 (84.5) | 0.046 |
| Presence of CMB, n (%) | 184/387 (47.5) | 2/4 (50) | 0.922 |

WMH: white matter hyperintensities; LIS: lacunar ischemic stroke; dICH: deep intracerebral hemorrhage; SVD: small vessel disease; PVS: perivascular spaces; BG: basal ganglia; CSO: centrum semiovale; CMB: cerebral microbleeds; SD: standard deviation.

**Table S4.** Comparison between patients with or without SWI/GRE sequence.

|  | **LIS with GRE/SWI (n=243)** | **LIS without GRE/SWI (n=591)** | ***P* value** | **dICH with GRE/SWI (n=148)** | **dICH without GRE/SWI (n=257)** | ***P* value** |
| --- | --- | --- | --- | --- | --- | --- |
| **Demographics** |  |  |  |  |  |  |
| Age, year, mean (SD) | 60.3 (10.9) | 62.3 (12.5) | 0.024 | 57.7 (12.6) | 57.8 (13.6) | 0.944 |
| Male sex, n (%) | 183 (75.3) | 420 (71.1) | 0.213 | 106 (71.6) | 186 (72.4) | 0.871 |
| **Vascular risk factors, n (%)** |  |  |  |  |  |  |
| Hypertension | 182 (74.9) | 421(71.2) | 0.283 | 114 (77.0) | 189 (73.5) | 0.436 |
| Diabetes mellitus | 89 (36.6) | 209 (35.4) | 0.73 | 20 (13.5) | 40 (15.6) | 0.576 |
| Hyperlipidemia | 75 (30.9) | 219 (37.1) | 0.089 | 14 (9.5) | 44 (17.1) | 0.034 |
| Coronary artery disease | 20 (8.2) | 24 (4.1) | 0.014 | 7 (4.7) | 9 (3.5) | 0.541 |
| Chronic kidney disease | 10 (4.1) | 82 (13.9) | <0.001 | 16 (10.8) | 29 (11.3) | 0.884 |
| Prior ischemic stroke | 32 (13.2) | 52 (8.8) | 0.057 | 7 (4.7) | 10 (3.9) | 0.685 |
| Prior ICH | 11 (4.5) | 12 (2.0) | 0.045 | 7 (4.7) | 17 (6.6) | 0.439 |
| Current smoking | 108 (44.4) | 238 (40.3) | 0.266 | 42 (28.4) | 82 (31.9) | 0.458 |
| Alcohol intake | 65 (26.7) | 172 (29.1) | 0.493 | 54 (36.5) | 101 (39.3) | 0.575 |
| **Pre-stroke medications, n (%)** |  |  |  |  |  |  |
| Antiplatelet | 35 (14.4) | 66 (11.2) | 0.193 | 10 (6.8) | 19 (7.4) | 0.811 |
| Lipid-lowering | 26 (10.7) | 54 (9.1) | 0.486 | 8 (5.4) | 16 (6.2) | 0.736 |
| **Admission blood pressure, mmHg,** **mean (SD)** |  |  |  |  |  |  |
| Systolic blood pressure | 154.1 (24.4) | 157.4 (22.2) | 0.062 | 171.5 (27.1) | 162.8 (27.4) | 0.002 |
| Diastolic blood pressure | 93.4 (15.6) | 91.9 (15) | 0.191 | 99.9 (15.8) | 97.3 (17.3) | 0.141 |
| **MRI markers of SVD** |  |  |  |  |  |  |
| Presence of lacune, n (%) | 146 (60.1) | 200 (33.8) | <0.001 | 57 (38.5) | 86 (33.5) | 0.306 |
| Total WMH score, median [IQR] | 3 [2–4] | 2 [1–4] | 0.05 | 3 [1–4] | 2 [1–4] | 0.901 |
| BG-PVS score, median [IQR] | 2 [1–2] | 2 [1–2] | 0.362 | 2 [1–2] | 2 [1–2] | <0.001 |
| Moderate-to-severe BG-PVS, n (%) | 149 (61.3) | 352 (59.6) | 0.638 | 105 (70.9) | 131 (51.0) | <0.001 |
| CSO-PVS score, median [IQR] | 2 [2–3] | 2 [2–3] | 0.639 | 2 [2–3] | 2 [2–3] | 0.110 |
| Moderate-to-severe CSO-PVS, n (%) | 210 (86.4) | 507 (85.8) | 0.811 | 138 (93.2) | 212 (82.5) | 0.002 |

SWI: susceptibility-weighted imaging; GRE: gradient-recalled echo; LIS: lacunar ischemic stroke; dICH: deep intracerebral hemorrhage; SVD: small vessel disease; WMH: white matter hyperintensities; PVS: perivascular spaces; BG: basal ganglia; CSO: centrum semiovale; CMB: cerebral microbleeds; SD: standard deviation.

**Table S5.** Number of voxels of the WMH probability distribution maps statistically significantly different in each patient group in terms of vascular risk factors and presence of CMB or lacunes. Numbers are considering significance at *P*<0.01 after FDR correction.

| Parameter | LIS patient group | | | dICH patient group | | |
| --- | --- | --- | --- | --- | --- | --- |
|  | Chinese Group template | Caucasian template | % max differences between templates | Chinese Group template | Caucasian template | % max differences between templates |
| Alcohol intake | 0 | 0 | 0 | 0 | 0 | 0 |
| Diabetes mellitus | 0 | 0 | 0 | 266 | 258 | 0.13 |
| Hyperlipidemia | 0 | 0 | 0 | 0 | 0 | 0.0035 |
| Hypertension | 0 | 3 | 6.88e-04 | 0 | 0 | 0 |
| Smoking status | 0 | 0 | 0 | 0 | 0 | 0 |
| Presence of CMB | 113115 | 140861 | 1.18 | 17536 | 20790 | 0.59 |
| Presence of lacunes | 91057 | 114368 | 2.18 | 2947 | 5186 | 1.13 |

CMB: cerebral microbleeds; FDR: false discovery rate; LIS: lacunar ischemic stroke; dICH: deep intracerebral hemorrhage.

Note: The percentage of maximum differences in the results using one template over the other are calculated with respect to the total number of voxels with non-zero values in the corresponding WMH probability distribution maps.

**References**

1. van Zagten M, Lodder J, Franke C, et al. Different Vascular Risk Factor Profiles in Primary Intracerebral Haemorrhage and Small Deep Infarcts Do Not Suggest Similar Types of Underlying Small Vessel Disease. *Cerebrovasc Dis* 1994; 4: 121–124.

2. Janssens E, Mounier-Vehier F, Hamon M, et al. Small subcortical infarcts and primary subcortical haemorrhages may have different risk factors. *J Neurol* 1995; 242: 425–429.

3. Schmal M, Marini C, Carolei A, et al. Different vascular risk factor profiles among cortical infarcts, small deep infarcts, and primary intracerebral haemorrhage point to different types of underlying vasculopathy. A study from the L’Aquila Stroke Registry. *Cerebrovasc Dis* 1998; 8: 14–19.

4. Beltrán I, Lago A, Tembl JI, et al. [Lacunar infarct and deep cerebral hemorrhage: a comparison of the risk factors]. *Rev Neurol* 1998; 27: 635–639.

5. Tsivgoulis G, Vemmos KN, Spengos K, et al. Common carotid artery intima-media thickness for the risk assessment of lacunar infarction versus intracerebral haemorrhage. *J Neurol* 2005; 252: 1093–1100.

6. Ronquillo JG, Rodríguez LJR, Rodríguez JC. Comparison among patient with lacunar cerebral infarct and deep primary intracerebral hemorrhages. *Acta Médica del Centro* 2007; 1: 4–9.

7. Labovitz DL, Boden-Albala B, Hauser WA, et al. Lacunar infarct or deep intracerebral hemorrhage: who gets which? The Northern Manhattan Study. *Neurology* 2007; 68: 606–608.

8. Cortina MG, Campello AR, Conde JJ, et al. Monocyte count is an underlying marker of lacunar subtype of hypertensive small vessel disease. *Eur J Neurol* 2008; 15: 671–676.

9. Kaplan EH, Gottesman RF, Llinas RH, et al. The Association between Specific Substances of Abuse and Subcortical Intracerebral Hemorrhage Versus Ischemic Lacunar Infarction. *Front Neurol* 2014; 5: 174.

10. Marsh EB, Gottesman RF, Hillis AE, et al. Predicting symptomatic intracerebral hemorrhage versus lacunar disease in patients with longstanding hypertension. *Stroke* 2014; 45: 1679–1683.

11. Morotti A, Paciaroni M, Zini A, et al. Risk Profile of Symptomatic Lacunar Stroke Versus Nonlobar Intracerebral Hemorrhage. *Stroke* 2016; 47: 2141–2143.

12. Lioutas V-A, Beiser A, Himali J, et al. Lacunar Infarcts and Intracerebral Hemorrhage Differences: A Nested Case-Control Analysis in the FHS (Framingham Heart Study). *Stroke* 2017; 48: 486–489.

13. Chen Z, Mo J, Xu J, et al. Risk Profile of Ischemic Stroke Caused by Small-Artery Occlusion vs. Deep Intracerebral Hemorrhage. *Front Neurol* 2019; 10: 1213.

14. Bernal M, Escarcena P, Arboix A, et al. Differential Characteristics of Ischemic and Hemorrhagic Stroke in Patients with Cerebral Small Vessel Disease. *Neurol India* 2021; 69: 85–90.

15. Muscari A, Masetti G, Faccioli L, et al. Association of Left Ventricular Hypertrophy and Atrial Fibrillation with Hemorrhagic Evolution of Small Vessel Disease. *J Stroke Cerebrovasc Dis* 2021; 30: 105946.

16. Wiegertjes K, Jansen MG, Jolink WM, et al. Differences in cerebral small vessel disease magnetic resonance imaging markers between lacunar stroke and non-Lobar intracerebral hemorrhage. *Eur Stroke J* 2021; 6: 236–244.

17. Goeldlin MB, Vynckier J, Mueller M, et al. Small vessel disease burden and risk of recurrent cerebrovascular events in patients with lacunar stroke and intracerebral haemorrhage attributable to deep perforator arteriolopathy. *Eur Stroke J* 2023; 8: 989–1000.

18. Dickie DA, Job DE, Rodriguez D, et al. Brain Imaging of Normal Subjects (BRAINS) age-specific MRI atlases from young adults to the very elderly (v1.0). Epub ahead of print 11 April 2016. DOI: 10.7488/ds/1369.

19. Dickie DA, Job DE, Gonzalez DR, et al. Use of brain MRI atlases to determine boundaries of age-related pathology: the importance of statistical method. *PLoS One* 2015; 10: e0127939.

20. Modat M, Ridgway GR, Taylor ZA, et al. Fast free-form deformation using graphics processing units. *Comput Methods Programs Biomed* 2010; 98: 278–284.

21. Clayden JD, Maniega SM, Storkey AJ, et al. TractoR: Magnetic Resonance Imaging and Tractography with R. *Journal of Statistical Software* 2011; 44: 1–18.

22. Valdés Hernández M, Grimsley-Moore T, Sakka E, et al. White Matter Hyperintensities Evolution Patterns 1 Year Post-lacunar Stroke and their association with post-stroke cognition. Epub ahead of print 23 June 2021. DOI: 10.7488/ds/3063.

23. Tang Y, Zhao L, Lou Y, et al. Brain structure differences between Chinese and Caucasian cohorts: A comprehensive morphometry study. *Hum Brain Mapp* 2018; 39: 2147–2155.

24. De Maesschalck R, Jouan-Rimbaud D, Massart DL. The Mahalanobis distance. *Chemometrics and Intelligent Laboratory Systems* 2000; 50: 1–18.

25. Jenkinson M, Bannister P, Brady M, et al. Improved optimization for the robust and accurate linear registration and motion correction of brain images. *Neuroimage* 2002; 17: 825–841.
